# Development of an interactive model for planning the care workforce for Alberta: case study

**DOI:** 10.1186/1478-4491-10-22

**Published:** 2012-08-20

**Authors:** Judy Bloom, Stephen Duckett, Andrea Robertson

**Affiliations:** 1 Workforce Planning, Alberta Health Services, Edmonton, Canada; 2 President and Chief Executive Officer, Alberta Health Services currently Professor, School of Public Health, University of Alberta and La Trobe University, Melbourne, Australia; 3 Health Professions and Chief Nursing Officer, Alberta Health Services, currently Chief Executive Officer STARS (Alberta Shock Transfer and Retrieval Service), Calgary, Canada

## Abstract

**Introduction:**

In common with other jurisdictions, Alberta faces challenges in ensuring a balance in health worker supply and demand. As the provider organization with province-wide responsibility, Alberta Health Services needed to develop a forecasting tool to inform its position on key workforce parameters, in the first instance focused on modeling the situation for Registered Nurses, Licensed Practical Nurses and health care aides. This case study describes the development of the model, highlighting the choices involved in model development.

**Case description:**

A workforce planning model was developed to test the effect of different assumptions (for instance about vacancy rates or retirement) and different policy choices (for example about the size of intakes into universities and colleges, different composition of the workforce). This case study describes the choices involved in designing the model. The workforce planning model was used as part of a consultation process and to develop six scenarios (based on different policy choices).

**Discussion and evaluation:**

The model outputs highlighted the problems with continuation of current workforce strategies and the impact of key policy choices on workforce parameters.

**Conclusions:**

Models which allow for transparency of the underlying assumptions, and the ability to assess the sensitivity of assumptions and the impact of policy choices are required for effective workforce planning.

## Background

Health services around the world face increasing challenges in matching the demand for needed skills with supply [1-3]. The creation of Alberta Health Services in May 2008 as the largest health care provider in Canada, a single entity responsible for providing hospital, continuing care services and public health throughout the Canadian province of Alberta (population 3.7 M) [4], caused a change in the focus of health workforce planning in the province.

The new entity had a number of inherent advantages compared to the prior structures:

• Because of its size, Alberta Health Services was able to create a specialist workforce planning function with the necessary expertise to undertake complex modeling;

• A new role, responsible for all health professions other than physicians, was created which provided provincial leadership in workforce innovation and role redesign, addressing a common barrier to the implementation of innovative workforce practices [5];

• Replacing nine separate regional health authorities and three specialist provincial authorities, Alberta Health Services was able to articulate a single provider view of provincial needs, rather than provider needs being the outcome of compromises between somewhat competing entities;

• As the province’s major provider entity, Alberta Health Services’ staffing choices largely determined demand for particular skill sets.

The last point was particularly important. Alberta Health Services’ approach to planning skill and resource requirements was developed during a time of relative fiscal constraint in Alberta following collapse of oil and gas prices [6]. Provincial government revenues are heavily dependent on oil and gas royalties which also collapsed, with a consequential reduction in the fiscal outlook for publicly-funded health services. Prior to the partial recovery in resource prices, Alberta Health Services had essentially been set a target of achieving a 10% reduction in spending levels. This required a thorough review of existing service delivery strategies, including examination of skill-mix issues.

Previous workforce planning attempts needed to reconcile competing views and policies of the predecessor organisations (nine regions and three provincial organisations) and was led by the ministry responsible for health care in Alberta (Alberta Health and Wellness) which had no direct levers to impact service delivery. A provincial approach to workforce planning was also difficult because of the impact of intra-provincial mobility: the rural regions were net importers of graduates from the universities and colleges located in the two main cities and those regions had different interests from the two city-based regions.

The universities and colleges responsible for producing the health workforce had ambiguous relationships with the local provider organizations [7]. Although some had good, formalized relationships, others had weak relationships with little input into curriculum design or intake policies. The main influence on university and college intakes was (and is) the provincial ministry responsible for funding the sector, Alberta Advanced Education and Training. The creation of Alberta Health Services allowed a single provider-voice to Advanced Education and Training so that the ministry was better positioned to ensure that university and college decision-making about intakes was consistent with directions of the provider organization.

The highest priority on Alberta Health Services’ workforce planning agenda was to plan for the nursing workforce, the largest employee group and a profession subject to past shortages in Alberta [8] and one particularly impacted by economic cycles [9]. Health spending in Alberta had increased faster than other Canadian provinces in the previous decade leading to higher per capita public spending, driven in part by higher input costs (e.g. more nurses per patient, higher pay for nurses) [10]. This also framed the workforce planning agenda, with the aim being a workforce plan which was sustainable into the long term.

The nursing workforce in Alberta is segmented into different categories of nurses: Licensed Practical Nurses (LPNs), Registered Nurses (RNs) and Registered Psychiatric Nurses (RPNs), with overlapping scopes of practice [11]. In addition, Alberta Health Services employs health care aides (HCAs) in a variety of roles. Typically, the workforce planning challenge is framed as balancing supply and demand in particular health professions, and although there are many potential variables which can affect balance [12], often the principal variable most easily within the control of decision makers is the size of intakes into college and university programs for the relevant profession. Workforce balance is the result of the interaction of supply and demand. Demand is not simply an exogenous variable related to population growth and ageing, it is also affected by workforce productivity and task/role assignment. Reliable systems to measure workload are still missing [13] which increases the contextual and subjective basis of estimates of care workforce requirements.

A study by Birch et al illustrated the relative impact of different strategies to achieve a balance in nursing workforce requirements in a number of Canadian provinces [14]. In their simulation study, compared to a baseline scenario where intakes into nursing programs would need to be increased by 2,975 places per year to avoid future shortages, if a productivity improvement of an additional 0.5% per year was achieved the required intake increase dropped to 825 places.

As Birch et al. point out:

"“productivity depends on a variety of factors including the intensity of work (proportion of paid hours given to patient care), how work is organised, technological inputs, and inputs of other types of professionals”."

It was this broader approach which was adopted by Alberta Health Services for its workforce planning endeavour, with a particular focus on examining different options for responding to changing service imperatives over the next decade. This inevitably means that there are a number of options which could be pursued and necessitates a workforce planning model which facilitates testing alternative scenarios [15-17].

As part of its strategic plan, Alberta Health Services had adopted values of respect, accountability, transparency and engagement. These values, particularly transparency and engagement, informed the approach adopted for care workforce planning.

### Case description: model design

Although the initial versions of the planning model were developed in Excel‘, the final model was developed in SAP BusinessObjects Xcelsius Enterprise‘. Development of the model involved the commitment of about nine person month’s of staff time, including gathering and collating input data.

The model values are the result of projections based on recursive equations incorporating constant and time variant values (see equations 1 & 2).

(1) $$\begin{array}{rl} \;E_{t+1}-E_{t} & =Number\;of\;Employees\;Demanded_{t}\\ & \;-Number\;of\;Employees\;Supplied_{\tau } \end{array}$$

(2) $$\begin{array}{rl} E_{t+1}-E_{t}= & \frac{\alpha }{AvgFTE}\{\left(E_{t}•\Delta Pop_{t}\right)\\ & +\gamma \left(\left[E_{t}•\Delta Pop_{t}\right]\;+\theta \left[New\;Graduates\right]\right)\}-\frac{1}{AvgFTE}\{E_{t}-\varphi \left(E_{t}\right)\\ & +\theta \left[New\;Graduates\right]+\in_{t}\} \end{array}$$

*E* = Employee Headcount

*t =* present year

α = proportion of unfilled positions – target vacancy rate

*Avg FTE* = Average Full Time Equivalency

*Δ Pop* = Population growth rate

*γ* = Additional casual requirement

*θ* = Percentage of graduates hired from Alberta post secondary institutions

φ = Percentage of employees ending employment with Alberta Health Services

∈ = System shocks (economic recovery and/or capital projects)

A critical early decision was about the scope of the model. The approach taken was to highlight and incorporate the potential for task and role substitution, so the model was designed to test various scenarios of different ways of addressing future service demand. In particular, the model incorporated supply and demand projections for the full “care workforce”, defined as RNs, RPNs, LPNs, and HCAs. Because of the small number of RPNs, they were included within the RN pool.

A second choice point related to what parameters or variables would be highlighted in various scenarios and what parameters would be implicitly held constant. The model facilitated evaluating the effects of the following variables:

• Skill mix (proportion of future demand to be met by RNs, LPNs and HCAs

• Separation (or staff turnover) rate for each group and retirement risks

• Vacancy rate

• Effect of economic recovery in Alberta

• Average FTE worked, including part-time to full-time ratio

• Number of graduates produced by Alberta universities and colleges and number of HCAs trained

• Proportion of graduates retained in Alberta

Development of the base-line scenario assumed maintenance of the status quo, including skill mix, retirement rates and the current pattern of hours worked per employee (full-time equivalent fraction, FTE).

Another choice point in the model was how to estimate future care. Several different approaches can be followed here with different ways of determining needs or demand [18]. As Dror et al. have pointed out in relation to broader health planning, needs and demand are overlapping concepts [19]. The need for future care is heavily influenced by growth and ageing of the population, mediated by the adopted service model. The approach adopted in modeling focused on the intersection of need and demand. The Alberta Health Services’ budget, which determines the number of staff who can be employed, is fixed for five years, growing in line with predicted inflation and growth and ageing of the population. That set a context to frame service growth, planned to grow in line with population growth and ageing, and consequential growth in care workforce demand.

Estimates of population growth for age groups were converted into sector-specific impacts based on historical patterns. (The “sectors” used were acute care, community/rural, seniors health, public health, primary care and mental health). The sectors were added to derive an “all sector” age-weighted population growth estimate (see Table 1).

**Table 1:** Projected increase in demand for care workforce, Alberta, 2010-2020

**Year**	2010	2011	2012	2013	2014	2015	2016	2017	2018	2019	2020
**Growth Rate**	2.56%	2.45%	2.40%	2.31%	2.24%	2.23%	2.24%	2.25%	2.27%	2.23%	2.25%

Almost every variable in the model involved some form of estimation or judgement to convert the available data into model parameters, including what data source to use as a basis for estimation of the current state. For example, available supply includes people working on a casual (hourly) basis. The only data sources to estimate availability of casual staff were the payroll systems of the previous entities (currently being integrated into a single payroll system). These payroll systems included all casual staff who worked any hours during the financial year, and so these data needed to be converted into expected hours to be worked and thus into FTE (see Appendix). Data from January 2010 for three former entities were analyzed to estimate the casual experience (other months were excluded because of holidays or the impact of Pandemic H1N1 2009, the latter inflating the typical use of casual staff).

### Baseline parameter estimates

Although the Alberta Health Services care workforce planning model was designed to allow modeling of different scenarios, the starting position or baseline needed to represent the status quo or unchanged policy assumptions. Again, this required estimation to provide a realistic baseline to judge different policy options.

The care workforce has been defined as the total FTE of the three occupation groups that deliver nursing and related services: RNs, LPNs, and HCAs. The December 2009 Alberta Health Services’ ratio of funded (budgeted) positions for these three groups was used as the baseline skill mix for forecasting demand for the majority of modeling scenarios (see Table 2).

**Table 2:** Full time equivalent (FTE) funded positions (‘budgeted FTE’), December 2009

	**Registered Nurses**	**Licensed Practical Nurses**	**Health Care Aides**	**All groups**
**Budgeted FTE**	14653	3060	3039	20753
**Percent**	70.6%	14.7%	14.7%	100%

The *separation rate* for each workforce group was estimated from 2009 payroll data: RNs 4.50%, LPNs 5.79%, and HCAs 7.66%. In parallel with the development of this model, Alberta Health Services implemented a vacancy management system which identified all funded positions within the organization and developed policies about authorizations necessary to fill vacancies. Calculation of a ‘vacancy rate’ relies on accurate estimation of both the numerator (vacancies) and the denominator (FTE) [20] with definitions of both varying [21]. The new vacancy management system was used to ensure consistency of definitions across the organization and calculation of a reliable estimate of budgeted FTE to identify vacancies. The expected *vacancy rate* was set in initial modeling at a normative rate of 3% for RNs, and 5% for LPNs and HCAs.

In addition to the indirect impact through provincial government revenues described above, the care workforce in Alberta is directly exposed to changes in the oil industry. In times of increased economic activity, a proportion of the workforce will withdraw their services, or reduce their hours worked in the health care system, as a result of higher relative hourly rates in the oil sector. The obverse occurs during an oil downturn. For example, when the Alberta economy experienced a downturn in 2008/2009, the major academic medical centre in Calgary, Foothills Medical Centre, was faced with an increased number of casual nursing care employees who wished to increase their worked hours, and/or who wished to transfer into permanent positions. The hypothesis incorporated into modeling scenarios contemplates the opposite effect occurring during a time of economic recovery. The Foothills Medical Centre experience in 2008/2009 provided the basis for an estimate of 6.63% of FTE held by the nursing workforce may be lost due to the impact of economic recovery, as nurses withdraw their services by reducing FTE or terminating employment. This impact was only applied to the Calgary, Edmonton and Fort McMurray workforce, the areas within the province most exposed to volatility in the energy sector.

Compared to all other Canadian provinces and territories, Alberta is the only province with a greater number of *part-time compared to full-time* employees. A transition to a greater number of full-time regular staff and a higher average FTE of part-time employees will reduce the actual number of employees required to fill the FTE.

Data for 2008 from the Canadian Institute for Health Information shows that Alberta had only 40.7% of Registered Nurses in a full-time position and 45.7% in a part time position (see Table 3). Nationally, the percentage of RNs in full-time positions is 58.1% with 31% in part-time positions. The same pattern is followed by both LPNs and RPNs.

**Table 3:** Proportion of workforce working as full-time, part-time or casual, Canada versus Alberta, by category 2007-2009

**Year**	**National**			**Alberta**		
	**Full Time**	**Part Time**	**Casual**	**Full Time**	**Part Time**	**Casual**
**RNs**						
**2007**	57%	32%	11%	40%	46%	14%
**2008**	58%	31%	11%	41%	46%	14%
**2009**a*				26%	45%	28%
**LPNs**						
**2007**	47%	35%	18%	41%	45%	14%
**2008**	49%	35%	16%	43%	43%	13%
**2009**^a^				29%	44%	27%
**HCAs**						
**2009**^a^				14%	46%	40%

^a^ 2009 data for Alberta Health Services only.

Data source for 2007 and 2008: Canadian Institute for Health Information.

The model implicitly assumed stability of intra-provincial flows by incorporating a single (controllable) variable for addition to the workforce supply: the graduation rate. The projected available supply of RNs and LPNs was calculated using the number of known graduates from the 2008–2009 year (provided by Alberta Advanced Education & Technology, the government department responsible for funding the university and college sector): 1582 for RNs and 802 for LPNs. Because universities and colleges in Alberta are net exporters of new graduates, these data were modified (reduced) to take account of the historical percentage of graduates who have been retained within Alberta: 70% for RNs, 90% for LPNs.

HCAs in Alberta are not directly regulated nor required to complete a defined program of study. Due to the inconsistent measurement available for HCA supply, the current supply was set to match current demand.

### Use of the model

The model was used in consultations with key stakeholders within Alberta including in a Discussion Paper [22] and presentations. A theme of the consultation process was about the need for change and the policy choices: that care needs could be met in a number of different ways with different consequences, for example, in terms of desirable intakes into university and college courses.

**Figure 1:**
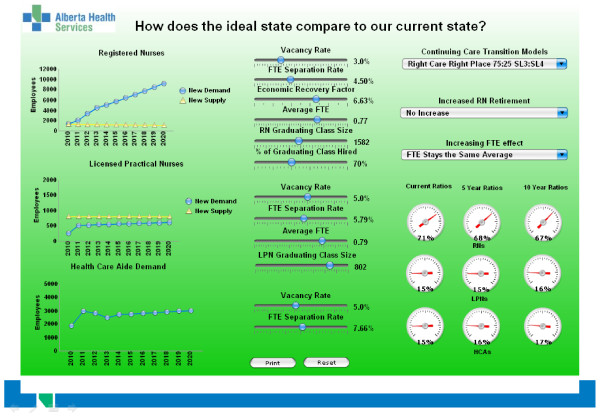
Example screen shot used in Alberta Health Services’ presentations to stakeholders as part of workforce panning, 2010.

The graphics capability of Xcelsius‘ was used extensively as a part of Powerpoint‘ presentations to stakeholder groups (see Figure 1).

The approach adopted in the presentations was one of total transparency in terms of assumptions and implications of model choices. The model made province-wide contemporary data available to stakeholders for the first time, building understanding of the implications of current policies.

Policy choices (e.g., size of graduating class) and assumptions (e.g., vacancy rate) were represented by levers in the middle column of the screen. In-built scenarios were in the third column. The model explicitly allowed the testing of changing demographics of the workforce (e.g., different retirement patterns) as an identified scenario, and modeling of different separation and recruitment patterns. The modeled effect of changes in these variables was represented by three graphs (in the first column) representing outcomes for the RN, LPN and HCA workforces. Shifts in any of the levers were reflected by changes in the graphs in real time, highlighting the impact of changes in choices or assumptions.

In contrast to the essentially infinite number of possibilities that could be modelled interactively in presentations, the Discussion Paper used six scenarios to represent the policy choices which could be considered:

1. Baseline (continuation of *status quo*)

2. Right care. Right place (a scenario based on changing the way aged care services are provided)

3. Full time work for full time pay (increasing average FTE worked)

4. Retirement impact – ageing workforce (changing assumptions about impact of retirements)

5. Right care, right place, right skill (version i, based on expansion of LPN workforce)

6. Right care, right place, right skill (version ii, based on expansion of HCA workforce)

The main body of the Discussion Paper summarized the outcome for each scenario in terms of care workforce supply and demand. An appendix table showing detailed model output was also presented for each scenario. Table 4 shows the typical tabular presentation using scenario 1 as an example.

**Table 4:** Projected increase in demand for care workforce, Alberta, 2010–2020, baseline scenario

**Year**	**RN**					**LPN**				**HCA**
	**RN Demand**	**RN Supply**	**Supply Surplus/ Deficit**	**FTE Vacancy Rate**	**% of Graduates Hired**	**LPN Demand**	**LPN Supply**	**Supply Surplus/ Deficit**	**% of Graduates Hired**	**Total HCA Demand**
2010	1381	1293	(88)	3%	70%	250	799	549	31%	1526
2011	1916	1107	(809)	5%	70%	620	799	179	77%	2669
2012	3244	1107	(2137)	9%	70%	632	799	167	79%	2733
2013	4481	1107	(3374)	13%	70%	642	799	157	80%	2794
2014	5163	1107	(4056)	14%	70%	651	799	148	81%	2856
2015	5865	1107	(4758)	15%	70%	665	799	134	83%	2923
2016	6591	1107	(5484)	17%	70%	680	799	119	85%	2991
2017	7342	1107	(6235)	18%	70%	694	799	105	87%	3059
2018	8120	1107	(7013)	20%	70%	710	799	89	89%	3130
2019	8909	1107	(7802)	21%	70%	723	799	76	90%	3194
2020	9725	1107	(8618)	22%	70%	738	798	60	90%	3264

Using the projections for RNs in 2012 as an example, Table 4 shows a demand projection for RNs of 3244 RNs in that year (based on the increase in service demand, the base-line assumptions about retirements and other departures, and the shortfall of 809 RNs from the previous year). Assuming recruitment of 70% of the Alberta RN graduations, this scenario would leave a shortfall of 2137 RNs in 2012. This gap should be addressed by a change in participation rates of Alberta RNs, change in recruitment patterns from outside the province (including international recruitment) or changed practice patterns to reduce the demand for RNs. If this gap is not addressed, and the base-line assumption in this scenario is that it is not, the shortfall of 2137 carries over to add to the demand estimate for 2013.

Modeling of scenario 1 shows a significant RN deficit which grows to more than 8500 RNs over ten years. Ongoing RN deficits would also lead to the RN vacancy rate growing to 22% in 2020 essentially because the graduation rate does not keep pace with growth in demand.

A particularly contentious component of workforce planning is assumptions about the planned skill mix for meeting future care needs. Two major reports on the future of health care in Canada both identified the need for role redesign as part of addressing workforce challenges [23,24] and this has to be at the forefront of any workforce planning. The Alberta Health Services’ model addressed this by the very model design: casting the problem in terms of care needs rather than a particular profession or professions and by modeling different skill mix scenarios.

The model was designed to allow exploration of a range of different choices in service design, intakes, etc. Under a number of the tested scenarios a shortfall in the care workforce was demonstrated. Such a shortfall could lead to gaps in service provision or a need to change recruitment strategies (e.g., to change intra-provincial flows or to recruit internationally). Variables to demonstrate the effects of these different policy choices were not incorporated in the model.

The Discussion Paper also tested the sensitivity of the model to changes in key assumptions or policies. Continuing the example of scenario 1, sensitivity analysis included adjusting separation (turnover) rates:

• Adjust RN separation rate from 4.50% to 3.43%;

• Adjust LPN separation rate from 5.79% to 4.16%;

• Adjust HCA separation rate from 7.66% to 5.93%;

Revised modeling to test model sensitivity showed that with these changed assumptions:

• the RN deficit is reduced from approximately 8,500 to 5,500 over ten years;

• the RN vacancy rate in 2020 is reduced to no more than 10%;

• the cumulative LPN surplus over 10 years grows from 1,784 to 3,160; and

• HCA demand over 10 years is reduced by approximately 1,500 HCAs.

## Discussion and evaluation

Health workforce planning is complex, involving multiple stakeholders: education and health sectors; industrial and professional organizations from the professions directly involved and from those who work with those professions; consumers; management and those responsible for funding the education and health sectors. The interests of the various stakeholders are not coincident and this, together with Alberta Health Services’ values, led to the adoption of a workforce planning approach which involved engagement of key stakeholders and was transparent in terms of potential policy choices and their consequences and in terms of the assumptions behind the workforce model.

There are multiple variables and policy choices involved in planning for any of the health professions and, because of scope overlap, contemporary workforce planning almost inevitably has to involve planning for separate professions and other staff groups, simultaneously. Together this means that workforce planning requires development of models to incorporate the interacting effects and to explicate policy and assumption choices using systems dynamics or other simulation approaches.

Alberta Health Services used proprietary software as the base for its model, partly chosen for the ease with which graphic presentations could be generated to be used in the consultation process.

Health workforce planning is particularly complex because of the long lead times associated with training health professionals. This has an important consequence: what might be best evidence and best judgement at a given time might be dramatically wrong at some time in the future as a result of unforeseen changes in the fiscal, policy or clinical environments (the global financial crisis impact being an example).

The track record of health workforce planning studies in getting projections right is not good [25]; a recent report prepared for a World Health Organization workshop on Global Workforce Strategy incorporates this damning indictment of the contemporary workforce planning experience: 

"“Health human resources planning in most countries has been poorly conceptualized, varying in quality, professions specific in nature, and without adequate vision or data upon which to base sound decisions” [26]"

Bloor and Maynard’s critique of physician workforce planning is probably also relevant to planning efforts for other professions as well:

"“The basis of current physician workforce planning is incomplete and mechanistic, using fixed ratio relationships that have no empirical validity” [27]."

New technology has meant that fixed ratio and other mechanistic approaches can now be easily replaced by modeling which allows the testing of different assumptions about staffing patterns, recruitment patterns and separation rates and the sensitivity of the assumptions involved in model development.

Visual presentation of the impact of different assumptions can facilitate feedback and model improvement, leading to more robust estimates of future demand and supply and a greater likelihood of achieving balance between supply and demand. It can also lead to a better shared understanding of the issues and the implications of policy choices, in turn facilitating consensus as to which options are viable. For example, as outlined above, the model described here highlighted the implications of current policies and demonstrated that continuation of current policies would lead to significant shortfalls in the care workforce and high vacancy rates, with the implication that the *status quo* was not a viable long-term option.

But the likelihood of model recommendations of a decade past, being appropriate a decade hence is trivially small. This means that workforce planning should not be seen as a once-off endeavour with the model applied and then forgotten. As with financial and other planning, the model should be reviewed at least annually, testing the validity of the assumptions and the model, and its recommendations fine-tuned appropriately. Some variables (e.g., proportion full-time) can and should be tracked on a more regular basis.

The test for any workforce planning model is thus not only whether the outputs from the model are accepted as reasonable and used as the basis for immediate action, but also whether the model can be used over time to inform refinement decisions. The Alberta Health Services’ model is proving useful on both fronts, and also the framework is being used for workforce planning for other professions.

The model has its limitations. First, even though the model involved planning for more than one discipline (RNs, LPNs and HCAs), the model implicitly assumed that the roles of other health professionals is held constant. Just as there is scope for task and role change between the care professions (as defined in this paper), there is also scope for task and role change involving nurses and other professions such as pharmacists [28,29]. Broader roles for other professions would impact (reduce) the demand for the care workforce being modeled.

Secondly, the model was technocratic in the sense that it assumed that demand for health care would grow on a technocratically rational basis. But health care everywhere (and Alberta is no exception) is subject to political influence be it tighter funding as a result of economic shifts or the alternative, greater than anticipated service expansion. These potential effects were not incorporated in the model.

Thirdly, model outputs were cast in terms of workforce impacts (numbers, vacancy rates, supply shortages). The model did not attempt a broader cost or economic assessment of the different scenarios or policy choices: What would be the training costs of different levels of intakes? What would be the impact of different staffing model choices on total service delivery costs? Health workforce planning choices are best not made in isolation from other aspects of health planning.

Finally, the model *per se* did not routinely incorporate any assessment of feasibility of modeled choices by, for example, presenting a dial showing the size of any modeled change from the baseline assumption. Any assessment of the difficulty of achieving a particular set of model choices was thus exogenous to the model.

## Conclusions

Contemporary workforce planning, such as that described here, needs to recognize the many competing variables that are simultaneously at play in shaping future workforce requirements. Workforce planning should not be simply based on extrapolating the current situation. Given those realities, and the very large number of possible options and interacting effects, modeling which easily allows assessment of options and different assumptions has to inform policy choices.

As with any workforce planning model, the Alberta Health Services model involved a number of assumptions which have been described. Transparency about assumptions is important to ensure model credibility. Emphasis in the Alberta Health Services approach was placed on ease of testing the impact of differences in policy choices to facilitate the use of the model as part of an engagement process.

Despite the limitations outlined above, the model developed by Alberta Health Services is now being used to influence budget decisions and advice to government about intakes into university and colleges in Alberta.

## Appendix A: Calculations for key supply variables

The following table shows the data definitions and sources for supply variables in Alberta Health Services (AHS) care workforce planning model (Table 5).

**Table 5:** Assumptions and key data sources

**Variable**	**Definition**	**Calculation**	**Data Source**
Total Active FTE	The assigned FTE an employee holds in the payroll systems. This is the employee's guaranteed minimum hours, as defined by their offer letter.	The assigned FTE held by all AHS employees in each occupation group, plus assigned FTE data provided by Covenant Health (a contractor to AHS). Assigned FTE includes all part time and full time employees who actually hold an FTE - casual employees are assigned an FTE of zero. Casuals are contemplated indirectly, as we know that a full time employee holding 1.0 FTE does not actually work 2022.75 hours per year - they are away from work due to vacation, sick, bereavement, etc. and we assume casual employees pick up shifts when full time employees are away from the workplace.	For AHS: Detailed Employee List – Interim HR database (Dec. 2009 data) For Covenant Health: Active FTE
Average FTE	The baseline average FTE is calculated to determine the current average FTE of those employees actually holding an FTE, not including casuals.	Total active FTE divided by Employee Count. (Employee count is the total number of employees holding an FTE, including employees holding part time, full time and temporary positions. It excludes the casual component of the workforce).	Detailed Employee List – Interim HR database (Dec. 2009 data) and Covenant Health data
	An average FTE proxy, inclusive of casual employees, has been calculated for HCAs employed in the private sector.		
		Total FTE in the Continuing Care sector divided by total HCA head count, including casuals. [7567 FTE / 17820 HCAs = 0.424 FTE]	
Casual Component	Estimated casual hours.	The casual component of the workforce can be captured in different ways. The number of casual employees on the payroll systems can be misleading, as many casual employees are active in the payroll systems, but not actively working shifts. A working casual proxy has been calculated, to help determine the size of the casual pool actually working shifts each month. (A separate casual component has not been factored into the continuing care estimates).	Detailed Employee List – Interim HR database (Jan. 2010 data)
		Active casual proxy = 52.6% of the total casual pool on the payroll system.	
		This proxy was calculated based on worked hours data pulled from former Capital, David Thompson, Northern Lights health regions, from January 2010 records.	
Registered Nurse (RN)	Registered Nurse (RN), Registered Psychiatric Nurse (RPN)*, Graduate Nurse, Clinical Instructors (within collective agreement only), Certified Graduate Nurse, Clinical Nurse Specialist (within collective agreement only), Assistant Head Nurse, Head nurse	Undergraduate Nurse, Nurse Practitioner (NP), Clinical Nurse Specialist (Out of Scope), Clinical Educators (Out of Scope)	Detailed Employee List – Interim HR database (Dec. 2009 data)
		*The RPN group is too small to be modeled separately, therefore, has been included in the RN group.	
Licensed Practical Nurse (LPN)	Licensed Practical Nurse, Orthopedic Technician, Operating Room Technician		Detailed Employee List – Interim HR database (Dec. 2009 data)
Health Care Aide (HCA)	Health Care Aide, Nursing Attendant, Home Care Aide	Therapy Aides, Therapy Assistants, Psychiatric Aides	Detailed Employee List – Interim HR database (Dec. 2009 data)

## Competing interests

The authors declare that they have no competing interests.

## Authors’ contributions

JB was responsible for the technical aspects of the model development described in this paper; all authors were involved in development of scenarios and assumptions, and writing and reviewing this paper.
